# Epidemiology of Carcinoma Breast in Young Adolescence Women

**DOI:** 10.7759/cureus.23683

**Published:** 2022-03-31

**Authors:** Sidra Latif, Sughra Perveen, Mazhar Iqbal, Tanweer Ahmed, Kulsoom Moula Bux, Syed Najib A Jafri

**Affiliations:** 1 Surgery, Jinnah Postgraduate Medical Centre, Karachi, PAK; 2 General Surgery, Jinnah Postgraduate Medical Centre, Karachi, PAK; 3 Data Analyst, Karachi School of Business and Leadership, Karachi, PAK

**Keywords:** triple-negative breast cancer, advanced breast cancer, breast lump, breast cancer, young adolescent women

## Abstract

Introduction

The aim of this study was to compare epidemiological characteristics of breast cancer in young adolescent women (YAW) versus older women (OW).

Methods

This was a cross-sectional prospective observational study, conducted in Ward 3, Jinnah Postgraduate Medical Center, Karachi, Pakistan, from September 2021 to February 2022. A total of 120 female patients were recruited in this study from the Outpatient Department of Jinnah Postgraduate Medical Center, out of which 22 patients were below the age of 40 years and 98 were above 40 years. For breast cancer diagnosis, we used the triple assessment method involving clinical examination, radiology, and histopathology. Diagnosed patients were further evaluated for hormonal status and metastatic workup. Results were noted on a performa, and differences between both age groups were analyzed.

Results

Out of 120 patients, 22 were younger than 40 years and 98 were older than 40 years. YAW used to present late after the appearance of symptoms. Patients of both age groups mostly presented with breast lumps (68.18% in YAW and 81.6% in OW). YAW presented with larger sizes of lumps and with more nodal involvement as compared to OW. BI-RADS IV (Breast Imaging Reporting and Data System Category IV) was the most commonly observed (27.27% in YAW and 48.97% in OW) mammographic finding in both age groups. Invasive ductal carcinoma was the most common histological type in both age groups (72.73% in YAW and 76.53% in OW). The triple-negative disease was more commonly found in YAW than OW (40.91% in YAW vs 21.43% in OW). We found that usually YAW presented at advanced stages (stages III and IV, 54.55%) and higher grades (grade III, 63.63%).

Conclusion

Breast cancer in young patients is rare but more aggressive with higher grades, advanced stages, and poor prognostic features. Heredity is mainly the risk factor in young breast cancer patients. There should be proper screening programs for high-risk group for early diagnosis and prompt treatment. Other age-specific concerns such as psychological impact of disease should be addressed as well.

## Introduction

Breast cancer is the most common type of cancer in females, accounting for 31.8% of all cancers [1]. Its incidence is increasing, particularly in low socioeconomic groups [2]. Breast cancer is common above the age of 40 years, and it is the commonest between 41 and 50 years [3]. Young women with breast cancer are those under 40 years of age [4]. The incidence rate in young adolescent women (YAW) is 5.6%, which increases rapidly due to screening mammography after 35 years of age. In comparison with the older age group, breast cancer in young patients is more likely to have familial predisposition genes, larger tumor size, unfavorable biological characteristics, advanced disease at diagnosis, and poor outcome. Chemotherapy and endocrine therapy are also different in young and older females. Breast cancer in young women is still uncertain with highly heterogeneous, aggressive, and complex biological features [5]. Breast cancer in YAW is difficult to manage due to the side effects of toxicity of different treatment strategies [6]. Young women have a longer time to live with side effects and complications of treatment and with risk of recurrence [7]. Moreover, young women with breast cancer are at a higher risk of psychological issues, and clinicians should deal with them to support patients during the lengthy diagnostic and therapeutic journey [8].

Age is not a predictor of chemosensitivity; the treatment choice is guided by the histological type, stage of the tumor, and patient's comorbidities [9]. Young females with breast cancer additionally have age-specific concerns such as infertility and physical appearance [10]. Pre-therapy fertility preservation options may be offered, which are currently available but potentially underutilized [11].

This study aimed to compare the epidemiology, clinicopathological characteristics, and prognostic features of breast cancer in females younger than 40 years versus older women with breast cancer.

The rationale of this study is to provide awareness about risk factors, clinical features, tools for diagnosis of breast cancer, and tumor behavior in young women so that early diagnosis and prompt treatment can be possible and increasing incidence of breast cancer can be reduced. Moreover, additional young age-related issues must be addressed.

## Materials and methods

This was a prospective cross-sectional observational study including patients who were presented to the symptomatic breast unit between September 2021 and February 2022 in Ward 3, Jinnah Postgraduate Medical Centre, Karachi, Pakistan. The patients were enrolled after obtaining ethical approval (approval number NO.F.2-81/2019-GENL/35813/JPMC) from the Institutional Review Board Committee of JPMC. A total of 120 patients were enrolled. Patients were classified into two groups: the first group included YAW less than 40 years of age and the second group included older women (OW) over 40 years of age. Patients’ sociodemographic, clinical data, physical examination features, diagnostic imaging modalities, and histopathology were assessed and analyzed using Statistical Package for the Social Sciences (SPSS) Version 25 (IBM Corp., Armonk, NY).

Collection of demographic and clinical data, and examination of patients were performed in the outpatient department. Age, area, marital status, number of children, breastfeeding, and family history were recorded in a performa.

Ultrasound in females below 35 years of age and mammography above 35 years were advised for radiological assessment. Mammographic findings of BI-RADS (Breast Imaging Reporting and Data System) category 0 were taken as inconclusive and further evaluated, BI-RADS I, II, and III as benign, BI-RADS IV and V as suspicious of malignancy, and BI-RADS VI as biopsy-proven carcinoma of the breast.

Suspected patients with breast carcinoma were confirmed by histopathological report, and results were recorded including histological type, hormonal status, and grade of the tumor. After the diagnosis of cancer, the metastatic workup included CT scan of the chest, CT scan of the abdomen, and bone scan were advised. We used the TNM classification for staging as per the American Joint Committee on Cancer (AJCC) Cancer Staging Manual, 8th edition and the molecular classification for hormonal receptor status.

## Results

Out of 120 patients, 22 (18.33%) were below the age of 40 years (range: 31-40 years), while 98 (81.66%) were above 40 years (range 40-80 years). YAW presented with an average period of one year after the appearance of symptoms, while OW presented at an average of 6 months. Among YAW, 22.7% of females had a family history of breast cancer, 13.64% were pregnant, and 9.09% were presented with bilateral breast involvement.

Patients of both age groups mostly presented with breast lumps followed by nipple discharge and locally advanced lesions. Other presentations are shown in Table 1.

**Table 1:** Clinical, radiological, and histopathological features of breast cancer in young versus older women BI-RADS, Breast Imaging Reporting and Data System

	Age < 40 years, n (%)	Age > 40 years, n (%)	Total, n (%)
Affected side			
Right	7 (31.81)	46 (46.93)	53 (44.16)
Left	13 (59.09)	52 (53.06)	65 (54.16)
Bilateral	2 (9.09)	0	2 (1.66)
Presenting complaints			
Lump	15 (68.18)	80 (81.63)	95 (79.17)
Nipple discharge	4 (18.18)	9 (9.18)	13 (10.83)
Mastalgia	0	2 (2.04)	2 (1.67)
Locally advanced (fungating/ulcerating lesion)	1 (4.55)	7 (7.14)	8 (6.67)
Bilateral	2 (9.09)	0	2 (1.67)
BI-RADS category			
0	0	6 (6.12)	6 (5)
IV	6 (27.27)	48 (48.97)	53 (44.17)
V	4 (18.18)	37 (37.55)	42 (35)
VI	2 (9.09)	7 (7.14)	9 (7.5)
Ultrasound suspicious	10(45.45)	0	10 (8.33)
Histopathology			
Invasive ductal	16 (72.73)	75 (76.53)	91 (75.83)
Invasive lobular	3 (13.64)	10 (10.20)	13 (10.83)
Inflammatory	1 (4.55)	6 (6.12)	7 (5.83)
Mucinous	0	3 (3.06)	3 (2.5)
Ductal carcinoma in situ	1 (4.55)	3 (3.06)	4 (3.33)
Paget’s disease	1 (4.55)	0	1 (0.83)
Others	0	1 (1.02)	1 (0.83)
Molecular subtype			
Luminal A	1 (4.55)	6 (6.12)	7 (5.83)
Luminal B	8 (36.36)	44 (44.9)	52 (43.33)
Basal cell like	9 (40.91)	21 (21.43)	30 (25)
HER2/neu enriched	2 (9.09)	23 (23.47)	25 (20.83)
Not applicable	2 (9.09)	4 (4.08)	6 (5)
Stage			
0	2 (9.09)	2 (2.04)	4 (3.33)
I	1 (4.55)	5 (5.10)	6 (5)
II	7 (31.82)	26 (26.53)	33 (27.5)
III	5 (22.73)	41 (41.84)	46 (38.33)
IV	7 (31.82)	24 (24.49)	31 (25.83)
Grade			
I	0	2 (2.04)	2 (1.67)
II	5 (22.73)	52 (53.06)	57 (47.5)
III	14 (63.63)	37 (37.76)	51 (42.5)
Not applicable	3 (13.64)	7 (7.14)	10 (8.33)

The left side is dominantly involved in both age groups. YAW presented with larger sizes of lump (average 8.54 cm) than OW (average 5.72 cm). Nodal involvement was present in 72.72% of women in the YAW group and 66.33% in the OW group.

Mammographic BI-RADS categories, histological and molecular types, and grading of tumor in both age groups are shown in Table 1.

## Discussion

The incidence of breast cancer increased in all age groups, particularly among women aged 20-39 years [12]. Breast cancer under the age of 40 years is found only in 7% but is the most common cancer in this female age group [13]. Different researchers have concluded that in breast cancer, young age is an independent poor prognostic factor [14]. In this study, 22 females were below 40 years of age. Around 26% of patients had a family history of breast or ovarian cancer [15]. Genotyping was traced in 12% of patients with pathogenic BRCA mutations [16]. A significant proportion of YAW with breast cancer carry germline mutations, showing the importance of prompt genetic testing for all young females at diagnosis [17]. Individualized screening, focusing only on some young high-risk women, can be beneficial. In our observation, 22.7% of patients had a family history of breast cancer, but due to limited resources, we did not conduct genotype testing in those patients.

Young symptomatic women with a lower likelihood of malignancy are at a significantly greater risk of delay in diagnosis of breast cancer [18]. We found that young females present later after the appearance of symptoms than older women.

With the help of clinical examination, breast cancer can be detected. Breast cancer usually presents with a lump (100%), lump with pain (5%), and nipple discharge (0.38%). A breast lump is the most common symptom [19]. However, occasionally nipple discharge is the earliest presentation of breast cancer. Mastalgia is a common presentation in breast clinics but mostly due to benign pathology. We also observed lumps as the most common presentation with no difference in terms of age.

In Langman et al.'s study, the mean size of all lesions on imaging in young cancer patients was 3.5 cm ± 2.9 cm [20]. Zouzoulas et al.’s study reported that 41% had T1, 28% had T2, 5.4% had T3, and 25.6% had T4 stage tumors [21]. We found larger tumor sizes in the young female age group.

Ultrasound helps to detect breast cancer specifically in the dense breast in patients up to 35 years of age and has an excellent diagnostic role in differentiation between benign and malignant lesions [22], with a sensitivity of 95.53% [23]. Mammography is a strong diagnostic tool for breast cancer detection in females above 35 years, screening purposes, and recurrence of breast cancer, with a sensitivity of 86.84% [24]. In our study, 10 patients were diagnosed by ultrasound and 110 by mammography.

In young patients with breast cancer, nodal infiltration was present in 73% and distant metastasis in 16% at the time of diagnosis [25], with multiple metastatic sites (28.3%) [26]. We found 72.72% lymph node involvement in young patients, and 31.82% presented with metastasized disease. Stage-wise differences between age subgroups are shown in Table 1.

The sensitivity of diagnosis of breast cancer is 100% for core biopsy. The most common histological type is invasive ductal (90%) [27]. The histological types we observed are shown in Table 1.

Compared to older women, early-onset breast cancer was found to have tumor grade 3 (29% in YAW vs 17% in OW), estrogen receptor negativity (45% in YAW vs 23% in OW), triple-negative (32% in YAW vs 10% in OW), and higher proliferation index Ki-67 (25% in YAW vs 10% in OW) [28]. Triple-negative breast cancer is characterized by poor prognosis due to high mortality and early relapse [29]. HER2/neu expression showed a statistically significant association with tumor stage [30]. Our study shows the difference in molecular classification in the young and older age groups in Table 1.

There were some limitations to the study. As it was performed in a single institute and with limited sample size and lack of diversity, the study is inappropriate for generalizing the findings to the whole population. We believe that more large-scale studies with a diverse sample size should be conducted to gain a more comprehensive understanding of the subject.

## Conclusions

Breast cancer in patients below the age of 40 years is rare but more aggressive than older females, with poor prognostic features. It is due to lack of awareness, unavailability of screening facilities, and overlapping of symptoms with changes in the breast during pregnancy and breastfeeding. The nonspecific symptoms and rare diseases in the younger age group are the cause of late presentation and advanced stages. Familial predisposition is more prevalent among young breast cancer patients and requires genetic counseling and early screening. We found breast cancer in young women to be associated with large tumor size, high-grade tumors, high incidence of nodal involvement, triple-negative molecular subtype, and need to be treated by a multidisciplinary team. Young women with breast cancer have age-specific concerns regarding changes in body image and fertility issues, which need special consideration.
